# Dietary changes among pregnant individuals compared to pre-pandemic: A cross-sectional analysis of the Pregnancy during the COVID-19 Pandemic (PdP) study

**DOI:** 10.3389/fnut.2022.997236

**Published:** 2022-12-01

**Authors:** Elnaz Vaghef-Mehrabani, Yanan Wang, Julia Zinman, Greis Beharaj, Marcel van de Wouw, Catherine Lebel, Lianne Tomfohr-Madsen, Gerald F. Giesbrecht

**Affiliations:** ^1^Alberta Children’s Hospital Research Institute, University of Calgary, Calgary, AB, Canada; ^2^Department of Pediatrics, University of Calgary, Calgary, AB, Canada; ^3^CSIRO Health and Biosecurity, Adelaide, SA, Australia; ^4^Department of Radiology, University of Calgary, Calgary, AB, Canada; ^5^Hotchkiss Brain Institute, University of Calgary, Calgary, AB, Canada; ^6^Department of Psychology, University of Calgary, Calgary, AB, Canada

**Keywords:** COVID-19 pandemic, dietary change, food categories, mental health, pregnancy

## Abstract

**Introduction:**

Dietary changes are common in pregnancy and may affect pregnancy outcomes, yet these changes and the associated contributory factors during the COVID-19 pandemic have been understudied. We aimed to investigate the association between dietary change and socioeconomic variables, pre-pregnancy BMI, and mental health symptoms; the change in intake of seven food categories and their reasons; and the association between intake of these food categories and mental health symptoms.

**Materials and methods:**

In this cross-sectional analysis, we used data from the Pregnancy during the COVID-19 Pandemic (PdP) cohort study that collected data from pregnant Canadian individuals (*n* = 9,870, gestational age ≤ 35 weeks) on socioeconomic factors, pandemic-related hardships, pre-pregnancy body mass index (BMI), dietary changes compared to pre-pandemic and the reasons for these changes. We assessed depressive and anxiety symptoms using the Edinburgh Postpartum Depression Scale (EPDS) and Patient-Reported Outcomes Measurement Information System (PROMIS)-Anxiety, respectively.

**Results:**

54.3% of the participants reported a change in their diet. Non-white ethnicity (OR = 1.33), job loss (OR = 1.29), clinically elevated depressive and anxiety symptoms (OR = 1.26 and 1.14, respectively), self-isolation (OR = 1.20), pre-pregnancy BMI (OR = 1.19), fear of COVID-19 (OR = 1.15), and pandemic phase at enrolment (OR = 0.90) significantly predicted dietary change. Most participants ate about the same amounts of dairy, meats and canned foods/dried goods as pre-pandemic (61.5, 61.7, and 60.2%, respectively), increased their intake of fresh vegetables/fruits and sweets/snacks (43.2 and 54.5%, respectively), and decreased fast-food and take-out/home delivery (53.2 and 43.1%, respectively). Changes in consumption of the food categories had a curvilinear association with mental health symptoms (except resilience) indicating greater symptoms with either decreased or increased intakes. Changes in craving, having more time for cooking/preparing foods, and being unable to go grocery shopping frequently (but not reduced affordability) were the main reasons driving these dietary changes.

**Conclusion:**

Some factors increase the odds of dietary change among pregnant individuals during the pandemic, with some changes toward a healthy and others toward an unhealthy diet. Given the importance of a healthy diet during gestation, identifying the risk and protective factors might be the first essential step in reducing the detrimental effects of unfavorable dietary changes during the pandemic on this vulnerable population.

## Introduction

A healthy diet during pregnancy is essential for meeting the changing metabolic needs of both pregnant people and their baby (1). Dietary changes are common in pregnant individuals, in part due to increased motivation for a healthier diet to positively impact their baby’s health (2). Other factors that might contribute to diet change during pregnancy include knowledge and skills on how to adopt a healthier diet, food aversions and cravings, time needed to prepare healthy foods, and finances (3). Although pregnancy-related dietary change is common during pregnancy, there can be little doubt that the global changes to everyday life imposed by the COVID-19 pandemic also contributed to dietary change.

Especially at earlier phases, pandemic-related restrictions imposed by governments around the world led to job losses and reduced wages, particularly in the lowest-income households (4). Worldwide, it also increased mental health symptoms (e.g., depression, anxiety, fear), particularly among pregnant individuals (5–8). Access to food, eating patterns and food choices were also altered during the pandemic, in large part due to pandemic-related restrictions and their socioeconomic consequences. Decreased access to sufficient and nutritious food, especially due to financial constraints, was one main reason for the dietary changes in the general population (9). In addition, increased stress was positively correlated with greater consumption of highly processed foods and alcohol to cope, added sugar intake, and food addiction symptoms (10). Stress may also have contributed to “out of control” eating during home confinement, and increased intake of caloric/salty foods and sweet and savory snack foods (11–13). Nevertheless, there is also evidence that some of these alterations were toward a healthier diet. Examples of positive changes include increased consumption of vegetables and fruits, and reduced intake of ready-made meals (13–16).

It is important to note that pandemic-related influences on diet have not been equal across all segments of the population. For example, individuals with higher body mass index (BMI) and lower age were more likely to increase their junk food intake and snacking frequency (17, 18). In addition, being of non-European decent and experiencing unfavorable changes to socioeconomic status (job loss, decreased income) increased the risk of food insecurity and decreased intake of healthy nutritious foods during the pandemic. Mental health problems (e.g., fear, depression, or anxiety) were also positively associated with both food insecurity and unhealthier food choices (19–23). The latter finding suggests that pregnant individuals might be more susceptible to dietary change during the pandemic, because physiological changes already pre-dispose them to mental health problems, and these problems can be exacerbated by high fear and stress widely experienced during the pandemic era (5, 24, 25).

Despite the importance of diet during pregnancy, only a few previous studies have examined pandemic-related changes in diet among pregnant individuals, and little attention has been given to the various inter-connected socioeconomic and mental health factors that may lead to these changes. A study in China found a higher tendency for emotional eating in people who lived in more severely affected areas, those who were more worried about the pandemic, and those with less physical activity. Higher emotional eating was associated with increased consumption of cereals and oil, and decreased intake of fish and seafood (26). Diet quality of pregnant individuals was also associated with the severity of the pandemic conditions in another Chinese study (27). According to a cross-national study, approximately one third of the pregnant individuals reported that the pandemic had at least moderately impacted their diet. Furthermore, there was a dose-response association with clinically elevated mental health distress, with higher depression/anxiety scores among those whose diet had been impacted the most (28). In an Italian study, about 44% of the pregnant participants reported that pandemic-related social restrictions gave them a chance to eat a healthier diet (29). Among pregnant people with diabetes in New Zealand, intake of most food items during the lockdown had no significant change compared to before, but participants reported eating more bread, and less battered fish, fries, and takeaways (30). These studies suggest that some pandemic-related dietary changes among pregnant individuals have been positive whereas others are likely to be detrimental. Pregnant individuals are a vulnerable group within the population, and it is essential that we understand how their dietary choices changed during the pandemic, and what factors may have contributed to these changes.

Our goal was to examine the ways in which pregnant people changed their diet during the COVID-19 pandemic. We used data from the ongoing Pregnancy During the COVID-19 Pandemic (PdP) study to (1) investigate the association between diet change and socioeconomic variables, pre-pregnancy BMI, and mental health symptoms; (2) determine the overall patterns of change in the intake of different food categories compared to pre-pandemic; (3) explore the pattern of association between intake of seven different food categories and mental health symptoms; and (4) examine the self-reported reasons for dietary changes.

## Materials and methods

We used data from the PdP cohort study to conduct this cross-sectional analysis. The PdP study recruited pregnant individuals from across Canada between April 2020 and April 2021. A protocol for the PdP study has been previously published (31). Research Electronic Data Capture (REDCap) (32) was used to collect and manage data that the participants provided on demographics, including socioeconomic status, health information, obstetric history, as well as diet changes and mental health symptoms/resilience through online surveys. This study was reviewed and approved by the University of Calgary Conjoint Health Research Ethics Board (REB20-0500), Calgary, AB, Canada. The participants provided their informed consent to participate in this study.

### Participants

Participants were included in this study if they were: living in Canada, ≥ 17 years of age, able to read and write in English or French, and ≤ 35 weeks’ gestation at the time of enrollment. There were no additional exclusion criteria for participation in this study. Recruitment was initiated through social media advertisements on Twitter, Facebook, and Instagram. We also targeted some of our ads to geographic regions with more socioeconomically disadvantaged neighborhoods to increase the number of individuals potentially most affected. No incentive was offered to the study participants.

### Study measures

#### Demographics and pandemic-related factors

As part of the enrollment survey, participants self-reported their ethnicity, education level, occupation, income, and marital status. Participants also disclosed COVID-19 infections and isolations, pandemic-related job loss and changes in income, as well as experience of food insecurity in the year preceding the pandemic (2019). They were considered food insecure if, in 2019, they reported that they had “often or sometimes experienced times that food did not last and they did not have money to buy more” or if “anyone in the household received food from a food bank, soup kitchen or other charitable agencies.” These two food security-related items were drawn from the National Population Health Survey (NPHS) conducted by Statistics Canada on a regular basis to collect data on the health of Canadians (33). Participants also provided gestational age as well as pre-pregnancy weight and height. Pre-pregnancy body mass index (BMI) was calculated as weight (Kg) divided by height squared (m^2^). Phase of the pandemic at study enrollment was also included as a factor that could affect study results. We divided the duration of the pandemic by 2 months, starting from April-2020.

#### Dietary changes and the related reasons

Participants completed a custom questionnaire developed for the PdP study to evaluate dietary changes during the pandemic compared to pre-pandemic. The diet questions were developed very early in the pandemic before anyone knew how the pandemic may be affecting diet. Therefore, the questions were designed to be descriptive and exploratory and focused on factors identified in previous studies as relevant to dietary change during pregnancy. First, participants were asked if they had changed their diet in any way compared to pre-pandemic. Those who answered “Yes” were then asked follow-up questions about how they changed their intake of seven food categories: fresh vegetables or fruits, dairy, meats, canned foods or dried goods, fast-foods, take-out or home delivery, and sweets or snacks. These food categories were selected based on an expert panel consensus on which foods would most probably be affected by the pandemic and which foods were most important to track during pregnancy. For each food category, participants responded on a 5-point Likert scale with one of the following options: “I eat much more,” “I eat more,” “I eat about the same,” “I eat less,” or “I eat much less.” Participants were asked to check off all the possible reasons for the changes they reported for each food category. These pre-specified options were: “Can no longer afford,” “Can’t go grocery shopping frequently,” Can spend more time cooking and preparing food,” “Change in craving” and “Other.” The first three options are predominantly pandemic-related reasons, while “Change in craving” is mainly a pregnancy-related reason.

#### Mental health symptoms and resilience

##### COVID-19 fear

Participants indicated their degree of COVID-19 fear by responding to the following three questions: (1) How much do you think your life is (was) in danger during the COVID-19 pandemic?; (2) How much do you think your unborn baby’s life is (was) in danger at any time during the COVID-19 pandemic?; and (3) How much are you worried that exposure to the COVID-19 virus will harm your unborn baby? For each of these questions, participants responded on a 100-point sliding scale, in which the left, middle, and right anchors indicated “Not at all” (0 points), “Somewhat” (50 points), and “Very much so” (100 points), respectively. Fear of COVID-19 was calculated as the mean z-scores for the three fear items. As previously reported, Cronbach’s alpha was 0.85, and a confirmatory factor analysis indicated excellent fit for a single factor, suggesting that the items comprise a single construct (5).

##### Depressive symptoms

Participants self-reported depressive symptoms within the past 7 days using the Edinburgh Postpartum Depression Scale (EPDS), which is reputable for its high reliability and validity and can be used to assess depressive symptoms during pregnancy (34, 35). The EPDS contains 10-items, each scored on a scale of 0–3. Overall scores ranged from 0 to 30, with higher scores indicating more severe symptoms of depression. Cronbach’s alpha for this measure was 0.88.

##### Anxiety symptoms

Participants recorded general anxiety symptoms within the previous 7 days using the Anxiety Adult 7-item short form of the Patient-Reported Outcomes Measurement Information System (PROMIS), which is noted for its reliability and validity in pregnancy (36). To calculate anxiety scores, we converted raw scores to T-scores, which range from 36.3 to 82.7, with a mean of 50 and a standard deviation of 10 according to US population data (37). Cronbach’s alpha was 0.93 for this measure (5).

##### Resilience

Participants self-reported their ability to cope with adversity using the 2-item Connor-Davidson Resilience Scale (CD-RISC 2), which has acceptable reliability and validity (38). The CD-RISC2 uses two items (“Able to adapt to change” and “Tend to bounce back after illness or hardship”) to quantify resilience and coping. Participants ranked their degree of coping on the two items using a 5-point Likert scale ranging from 0 (“not true at all”) to 4 (“True all the time”). Scores ranged from 0 to 8, with higher scores indicating greater resilience and successful coping.

### Statistical analyses

Data were screened and cleaned prior to analyses to ensure every record was valid. We used SPSS 26.0 software to analyze the data. For multiple comparisons and regression analyses, we used the Benjamini-Hochberg procedure to correct for the false discovery rate (FDR) of 5%. Descriptive analyses were used to characterize the study population. For the quantitative data (maternal age, gestational age, pre-pregnancy BMI, and mental health symptoms/resilience), Kolmogorov-Smirnov test were conducted to check for normality of distribution. If the data was normally distributed, we reported Mean and standard deviation (SD) for the variable. Otherwise, median and percentiles 25 and 75, were presented. We presented qualitative (binary or ordinal) data as number (n) and percentage. We conducted Analysis of variance (ANOVA) and Chi-Square tests to compare all the study variables, between the analytic sample and those who were excluded from analyses.

*Aim 1:* To investigate the association of diet change with socioeconomic variables, pre-pregnancy BMI, and mental health symptoms/resilience we used univariable binary logistic regression. We followed with a multivariable binary logistic regression to determine the unique contribution of each predictor in a model that included all the variables that were significantly associated with diet change in the univariable models. We used the z-scores of the mental health symptoms/resilience in the regression models to allow for comparison of effects across mental health indicators.

*Aim 2:* We used descriptive analyses to describe the overall patterns of change in the intake of the seven food categories compared to pre-pandemic.

*Aim 3:* To explore the pattern of association between intake of each of the seven food categories and mental health/residence indicators, we used Analysis of covariance (ANCOVAs) to estimate the marginal means for each of the five change categories for each of the seven food categories, after controlling for relevant covariates. Since education, income change, food insecurity in the preceding year (2019), and phase of the pandemic at study enrollment are potential confounding factors that might affect both dietary intake and mental health/resilience during the pandemic, we controlled for these variables in the analyses.

*Aim 4:* We used descriptive analyses to examine the self-reported reasons for dietary changes in the seven categories based on the following response options: “Can no longer afford,” “Can’t go grocery shopping frequently,” “Can spend more time cooking and preparing food,” “Change in craving,” and “Other.”

## Results

### Demographics of the study population

A total of 10,850 pregnant individuals enrolled in the PdP study and provided at least partial responses on the baseline survey. Of these, 9,870 participants answered the questions on dietary change and were included in the analyses. Comparison of baseline characteristics between the participants who were included in the analyses and those who were not (*n* = 980), showed that those excluded were about the same age (31.2 versus 31.9 years), a lower proportion had university degrees (49.8 versus 65%), fewer had household annual income above $100,000 (46.5 versus 59.1%), and fewer had a full-time job (72.5 versus 76.7%). There was no significant difference between the two groups for gestational age at survey, pre-pregnancy BMI, ethnicity, and marital status (see Supplementary Table 1).

Table 1 presents the socioeconomic characteristics, pre-pregnancy BMI, and mental health symptoms of the study participants. Almost half (48.6%) of the population was overweight (BMI between 25 and 29.99) or obese (BMI ≥ 30). Most participants self-identified as white/Caucasian (83.5%), reported having completed a trade/technical degree or higher (90.1%), were married or cohabited with their partner (95.2%) and worked full-time (76.1%). More than 75% of the individuals had a household annual income equal to or over $70,000 in the year preceding the pandemic, and more than 40% reported a decrease in their income due to COVID-19 pandemic. About 70% of the participants were recruited to the study during the first 6 months of the pandemic.

**TABLE 1:** Socioeconomic characteristics and mental health symptoms/resilience of the study participants (*n* = 9,870).

Variables	Mean (SD)
Age (years)	31.86 (4.39)
Missing (n)	49
Gestational age at survey (weeks)	20.53 (8.70)
Missing (n)	0
Pre-pregnancy BMI (Kg/m^2^)	26.24 (5.85)
Missing (n)	397
Fear of COVID-19	−0.002 (0.88)
Missing (n)	119
Depression	10.19 (5.49)
Missing (n)	420
Anxiety	58.13 (8.34)
Missing (n)	452
Resilience	5.84 (1.35)
Missing (n)	1485

	***n (%)***

**Ethnicity**	
White (Caucasian)	8239 (83.5)
Other ethnic groups	1494 (15.1)
Missing	137 (1.4)
**Education**	
Professional (MD, JD, DDS, ETC)	453 (4.6)
Doctorate (PhD)	298 (3.0)
Masters’ degree	1800 (18.2)
Bachelor’s degree	3843 (38.9)
Completed trade/Technical degree	25.03 (25.4)
Completed high school diploma	808 (8.2)
Less than high school diploma	119 (1.2)
Missing	46 (0.5)
**Job status**	
Working full-time	7515 (76.1)
Working part-time	1116 (11.3)
Unemployed/Laid off	206 (2.1)
Looking for work	111 (1.1)
Keeping house/Raising children	848 (8.6)
Retired	2 (0.0)
Missing	72 (0.7)
**Household income**	
≥ $200,000	952 (9.6)
175,000–$199,999	624 (6.3)
150,000–$174,999	1086 (11.0)
125,000–$149,999	1286 (13.0)
100,000–$124,999	1844 (18.7)
70,000–$99,9999	1949 (19.7)
40,000–$69,9999	1296 (13.1)
20, 000–$39,999	545 (5.5)
< $20,000	218 (2.2)
Missing	70 (0.7)
**Marital status**	
Married/Cohabiting	9397 (95.2)
Other	428 (4.3)
Missing	45 (0.5)
Job loss (yes)	1609 (16.3)
Missing	1239 (12.6)
**Income change**	
Substantially decreased	1368 (13.9)
Somewhat deceased	3029 (30.7)
Not change	4764 (48.3)
Somewhat increased	606 (6.1)
Substantially increased	43 (0.4)
Missing	60 (0.6)
Food insecurity (yes)	848 (8.6)
Missing	74 (0.7)
Self-isolation (yes)	4496 (45.6)
Missing	0 (0)
**Phase of the pandemic at study enrolment**	
April–May	4687 (47.5)
June–July	1888 (19.1)
August–September	478 (4.8)
October–November	1079 (10.9)
December–January	1130 (11.4)
February–March	530 (5.4)
April	78 (0.8)
Missing	0 (0)

### Aim 1: Association of socioeconomic characteristics, pre-pregnancy body mass index, and mental health indicators/resilience with dietary change

Among those who responded to the diet questions (*n* = 9,870), 54.3% reported a change in their diet.

As shown in Table 2, socioeconomic factors that were associated with increased odds of dietary change after Benjamini-Hochberg correction included higher pre-pregnancy BMI, identifying with a racial/ethnic group other than White, job loss, decreased income, food insecurity in the year preceding the pandemic (2019), and self-isolation due to COVID-19. In contrast, a higher level of education and the later stages of the pandemic were associated with decreased odds of dietary change. Maternal age, gestational age, and household income in the year preceding the pandemic (2019) were not associated with dietary change.

**TABLE 2:** The association between demographic characteristics, pre-pregnancy body mass index (BMI) and mental health symptoms/resilience (predictors), and dietary change.

Predictors	Unadjusted model^†^			Adjusted model^‡^		
	OR	95% CI	*P*	OR	95% CI	*P*
Age	1.00	0.99, 1.01	0.780	−	−	−
Gestational age at survey	1.00	0.99, 1.00	0.934	−	−	−
Pre-pregnancy BMI	1.21	1.16, 1.26	< 0.001*	1.19	1.13, 1.25	< 0.001*
Fear of COVID-19	1.41	1.35, 1.48	< 0.001*	1.15	1.08, 1.22	< 0.001*
Depression z-scores	1.54	1.47, 1.61	< 0.001*	1.26	1.16, 1.38	< 0.001*
Anxiety z-scores	1.51	1.45, 1.58	< 0.001*	1.14	1.04, 1.24	0.003*
Resilience z-scores	0.84	0.80, 0.87	< 0.001*	0.99	0.94, 1.04	0.745
Ethnicity	1.43	1.28, 1.60	< 0.001*	1.33	1.15, 1.54	< 0.001*
Education	0.95	0.92, 0.98	0.002*	1.02	0.97, 1.06	0.432
Household income	1.04	0.40, 2.72	0.929	−	−	−
Job loss	1.43	1.28, 1.60	< 0.001*	1.29	1.12, 1.49	< 0.001*
Income change	1.16	1.11, 1.22	< 0001*	0.98	0.91, 1.04	0.486
Food insecurity	1.56	1.34, 1.80	< 0.001*	1.11	0.88, 1.38	0.341
Self-isolation	1.46	1.35, 1.58	< 0.001*	1.20	1.09, 1.33	< 0.001*
Phase of the pandemic at study enrolment	0.91	0.89, 0.93	< 0.001*	0.90	0.88, 0.93	< 0.001*

^†^Results based on Univariable logistic regression.

^‡^Results based on Multivariable logistic regression including only the predictors with significant results from the Univariable logistic regression models.

*Significant after Benjamini-Hochberg correction.

Diet change during the pandemic was also associated with mental health symptoms. For every 1 SD increase in depression symptoms, anxiety symptoms, or fear of COVID-19, the odds of dietary change increased by 54, 51, and 41% respectively. Resilience was negatively associated with diet change; the odds of diet change decreased by 16% for every 1 SD increase in resilience.

Having identified individual factors associated with dietary change, we conducted multivariable logistic regression to determine the unique contribution of each predictor in a model that included all factors that were significantly associated in the univariable analyses. As shown in Table 2, ethnicity (OR = 1.33), job loss (OR = 1.29), and depression (OR = 1.26) had the greatest unique association with dietary change, followed by self-isolation (OR = 1.20), pre-pregnancy BMI (OR = 1.19), and fear of COVID-19 (OR = 1.15). Associations were no longer significant for resilience, education, income change, and food insecurity in the multivariate model, indicating that these factors likely contributed to diet change through the other factors in the model with no unique effect of their own.

### Aim 2: Examining changes in consumption of seven categories of foods

Those who reported that their diet had changed during the pandemic (*n* = 5,359) were asked to indicate whether their consumption of food in seven food categories (fresh vegetables and fruits, dairy, meats, canned foods and dried goods, fast-food, take-out/home delivery, and sweets and snacks) increased or decreased. Some participants reported that they did not consume foods in one or more of the seven food categories [Fresh vegetables and fruits, *n* = 6 (0.1%); Dairy, *n* = 92 (1.7%); Meats, *n* = 140 (2.6%); Canned foods and dried goods, *n* = 131 (2.4%); Fast-food, *n* = 125 (2.3%); Take-out/home delivery, *n* = 92 (1.7%); and sweets and snacks, *n* = 35 (0.7%)].

**FIGURE 1:**
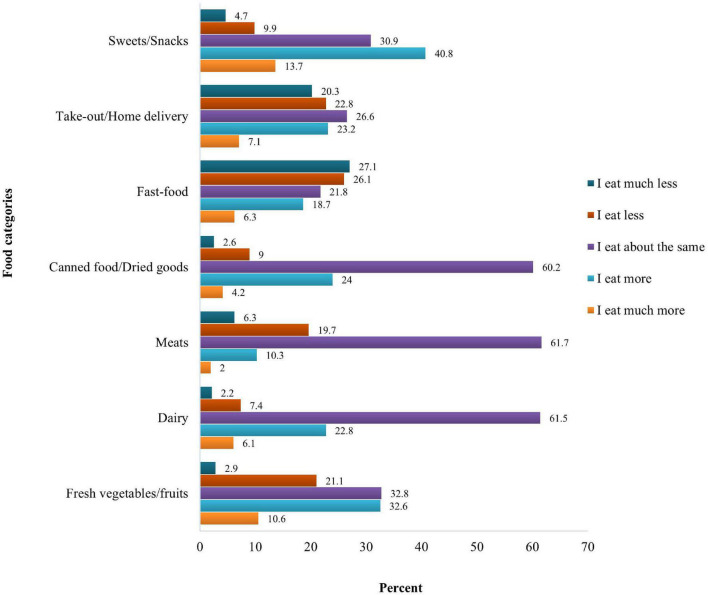
Dietary changes among pregnant individuals during COVID-19 pandemic compared to pre-pandemic.

Figure 1 displays changes in participant food consumption during COVID-19 compared to the pre-pandemic. Even though these analyses were conducted among participants who indicated that they had changed their diet, the most frequently chosen option across the food categories was “I eat about the same.” This was especially the case for dairy, meats, and canned foods/dried goods where more than 60% of the participants reported eating about the same amount. Among those who reported a change in their consumption of these three food categories, most individuals increased their intake of dairy and canned foods/dried goods (28.9 and 28.2%, respectively), while most of them decreased consumption of meats (26.0%). The majority of participants increased their consumption of fresh vegetables and fruits (43.2%), and about a third (32.8%) reported eating about the same. The largest increase in consumption was seen for sweets and snacks with 54.5% reporting a much more or more intake, while only 14.6% reduced their consumption of the food category. The largest decreases were observed for fast-food and take-out/home delivery, with more than half (53.2%) reporting less fast-food and 43.1% reducing take-out/home delivery.

### Aim 3: Associations between changes in consumption of food categories and mental health symptoms/resilience

As summarized in Table 3, there was a significant association between the mental health indicators and changes in intake of all the seven food categories. Resilience was associated with changes in intake of fresh vegetables and fruits, canned foods/dried goods, fast-food, take-out/home delivery and sweets/snacks.

**TABLE 3:** The association between mental health and resilience indicators and changes in intake of seven food categories.

Variables	Fresh vegetables and fruits	Dairy	Meats	Canned foods and dried goods	Fast-food	Take-out and home delivery	Sweets and snacks
Fear of COVID-19	(4, 5245), 10.02*	(4, 5161), 3.28*	(4, 5115), 6.41*	(4, 5123), 8.27*	(4, 5128), 11.04*	(4, 5163), 19.71*	(4, 5219), 5.51*
Depression	(4, 5096), 32.62*	(4, 5015), 17.66*	(4, 4968), 21.12*	(4, 4979), 24.63*	(4, 4981), 14.29*	(4, 5014), 9.90*	(4, 5071), 13.40*
Anxiety	(4, 5074), 26.99*	(4, 4993), 8.11*	(4, 4947), 11.40*	(4, 4959), 20.23*	(4, 4960), 7.41*	(4, 4993), 10.59*	(4, 5051), 6.14*
Resilience	(4, 4550), 7.88*	(4, 4486), 0.72	(4, 4435), 1.45	(4, 4449), 3.62*	(4, 4445), 4.89*	(4, 4479), 6.65*	(4, 4530), 4.82*

Results are based on ANCOVA, comparing the five categories of responses, and including education, income change, food insecurity in the preceding year (2019), and phase of the pandemic at study enrolment as covariates; Data are presented as (df) F.

*Significant after Benjamini-Hochberg correction.

**FIGURE 2:**
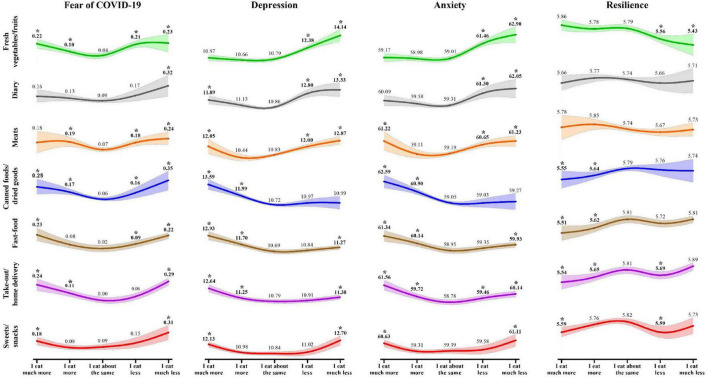
Estimated marginal means of mental health indicators relative to dietary change in seven food categories.

As shown in Figure 2, changes in consumption of the seven food categories had a curvilinear and largely U-shape association with mental health symptoms indicating greater symptoms of psychological distress with larger dietary changes (either decreased or increased intakes). There was a J shape association between depressive symptoms and changes in dairy intake, and between fear of COVID-19 and sweets/snacks intake, suggesting that decreased intake was associated with higher scores of mental health indicators compared to increased consumption. Both depressive symptoms and anxiety had a backward-J shape association with fast-food and take-out/home delivery, indicating higher scores of mental health indicators in those who increased their intake than those who decreased. It is noteworthy that only a linear pattern was observed between depression or anxiety and fresh vegetables and fruit consumption, where higher mental health symptoms were associated with decreased vegetables and fruit consumption. Similarly, for canned foods and dried goods, only increased consumption was associated with elevated depression and anxiety symptoms. Overall, these findings suggest that both the magnitude of change and the direction of change in dietary intake are associated with relatively elevated depression and anxiety symptoms.

In contrast to the curvilinear pattern of results for the mental health symptoms, the associations between resilience and changes in intake were relatively linear with few significant associations. Changes in meat and dairy consumption were not related to resilience at all. Consuming fewer fresh vegetables and fruits, and increased consumption of canned foods/dried goods, fast food, take-out/home delivery and sweets/snacks were associated with decreased resilience. Eating somewhat less take-out/home delivery and sweets/snacks was also associated with reduced resilience. No pattern of consumption was associated with increased resilience.

### Aim 4: Self-reported reasons for change in intake of seven food categories

**FIGURE 3:**
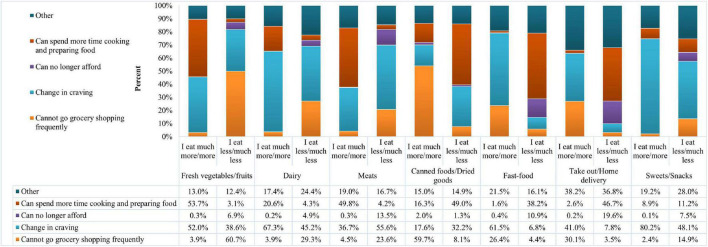
Self-reported reasons for change in consumption of the seven food categories compared to pre-pandemic.

Figure 3 illustrates the reasons reported by the participants for the changes they made to their intake of the seven food categories compared to pre-pandemic. As expected, change in craving was a major pregnancy-related reason for changes in some food categories; specifically, it was the top reason for increased intake of fast-food and take-out/home delivery, decreased intake of meats, and both decreased and increased consumption of dairy and sweets/snacks. Among the pandemic-related reasons for change, not being able to afford specific foods was a minor reason whereas the ability to spend more time cooking and not being able to go grocery shopping frequently were major reasons for diet change. “Being able to spend more time on cooking and preparing foods” was the top reason for increased consumption of fresh vegetables/fruits and meats, and decreased intake of canned foods/dried goods, fast-food, and take-out/home delivery. “Not being able to go grocery shopping frequently” was the top reason for decreased intake of fresh vegetables/fruits and increased intake of canned foods/dried goods.

## Discussion

### Dietary changes and the associated factors

About half of our study participants changed their diet. This is in keeping with an Italian study which reported that 44% of pregnant and recently delivered individuals claimed that the pandemic gave them the chance to eat healthier (29) and a global study across 12 countries which reported that 61.4% of pregnant individuals indicated that the pandemic had impacted their diet at least “a little bit” (28). Despite the pervasive and sustained nature of the disruption to everyday life caused by the pandemic, a large proportion of pregnant individuals were able to maintain their usual diet. Even among those who reported that their diet changed in some way, the most frequently reported change across the seven food categories was “eating about the same,” suggesting that pandemic-related dietary changes were more specific than general for most people. The pandemic seems to have also affected specific groups of pregnant individuals more than others. Specifically, more dietary changes were observed among those with non-white ethnicity, those who lost their job, those with higher pre-pregnancy BMI, greater fear of COVID-19, higher depressive and anxiety symptoms, those who self-isolated due to COVID-19, and those who were recruited in the earlier phases of the pandemic.

#### Socioeconomic factors

Non-white ethnicity increased the odds of dietary change during the pandemic (20). In the study by Choi et al., among pregnant individuals, about 40% of the participants were white (28), which might partially explain the higher rate of dietary change in that study compared to ours (61.4 vs. 54.3%). Additionally, higher education was associated with lower odds of dietary change in our univariable regression model but lost its significant association in the multivariable model. This might imply that higher education does not independently predict dietary change but is associated with other direct predictors like job loss or income. The evidence shows that individuals especially females with lower education were more likely to lose their jobs during the pandemic compared to those with a college or university degree (39). Our data is in line with this evidence as individuals with higher education were about 40% less likely to be laid off (95% CI 0.57, 0.64; *P* < 0.001).

Our data regarding food insecurity pertained to the year preceding the pandemic (2019) and might not directly and accurately demonstrate food insecurity during the pandemic. Moreover, food insecurity was reported only in 8% of our study population, which was lower than the other studies (40). It is also noteworthy that food insecurity decreased during early lockdown in Canada, unlike other countries (14), possibly due to the government supports and increased net income especially among low-income households (4).

#### Pre-pregnancy body mass index

The significant association we observed between BMI and dietary change is supported by previous research (13, 17, 18). People with overweight or obesity are more likely to exert problematic eating behaviors like overeating or eating while not hungry (41). The lockdown that followed COVID-19 outbreak might have promoted these eating behaviors by exposing people to increased access to stocked food (18). Since both pregnant individuals and those with higher BMI are at greater risk for COVID-19 complications (42), it may be reasonable to encourage those with higher BMI to adopt healthy eating patterns during the pandemic.

#### Psychological factors

We showed in our previous reports on this cohort that anxiety and depression symptoms are elevated among pregnant individuals in our population compared to the pre-pandemic cohorts (8), and that fear of COVID-19 was associated with increased odds of scoring above the clinical cut-off on both anxiety and depression measures (5). Since mental health and dietary practices have bidirectional association, different studies have approached the association from either direction based on their hypotheses. Choi et al., reported that the odds of mental health distress (combination of depression and anxiety) were higher by 4.72 times in those with diet change (28). Emotional eating was observed in a retrospective study among new mothers who experienced the COVID-19 lockdown in their third trimester, especially those who lived in Wuhan and surrounding provinces (26). Emotional eating is defined as changes to eating behavior ranging from over/binge eating to strict calorie restriction in response to emotions including anxiety, depression, fear, etc., (43).

#### Pandemic-related factors

Two pandemic-related factors that might affect dietary change are self-isolation and phase of the pandemic. Self-isolation was significantly associated with dietary change in our study. It can impact eating behaviors and patterns in both positive and negative ways through decreased access to fresh produce, emotional eating resulting from boredom, and allowing people to spend more time on cooking and preparing foods (17, 26, 44).

Most of the previous studies on dietary changes during the pandemic have recruited participants over a short period of 2–3 months. Therefore, they were unable to investigate whether those recruited months after the pandemic began differed from those recruited earlier in terms of dietary change. The study by Choi et al., was conducted in May–June 2020 when people were highly affected by the pandemic breakout (28), while our study recruited pregnant participants throughout different phases of the pandemic.

### Changes in intake of seven food categories and self-reported reasons for change

Overall, most participants consumed about the same of most food categories (especially dairy, meats, and canned foods/dried goods) as pre-pandemic. Among those who changed their intake, most increased their consumption of fresh vegetables/fruits, dairy, canned foods/dried goods, and sweets/snacks, and decreased intake of meats, fast-food, and take-out/home delivery. Changes in craving, having more time to spend on cooking and preparing foods, and not being able to go grocery shopping frequently were the main reasons driving these dietary changes in our study.

In a study by Zhang et al., more than half of the participants reported eating about the same of most food groups as pre-pandemic. The authors observed the greatest decrease in consumption of fish, sugar/honey, fats, meats, pulses, legumes and nuts, and the greatest increases in consumption of vegetables and fruits, eggs, dairy, and cereals (26). Our findings were comparable to those of Zhang et al., as we also found that more people decreased intake of meats, and increased consumption of fruits and vegetables and dairy. An online survey on pregnant individuals with diabetes showed that other than increased bread, and decreased battered fish, hot chips/fries and takeaways, the pandemic had no effect on consumption of most food groups (30). Similar to Amataiti et al., we found decreased take-out intake. But our findings were not in agreement with that study as we also found a greater percentage of people changing their intake of some other food categories. This discrepancy might have been a result of how the dietary data were collected (more quantitative in that study compared to ours, with statistical analysis performed to test the changes). Moreover, pregnant individuals with diabetes might have been more careful and cautious with their dietary intake during the lockdown to avoid complications that could lead to medical care requirements. Neither of the previous two studies among pregnant individuals explored the possible reasons for changes in intake of each food group.

The majority of our participants ate about the same dairy and meats as pre-pandemic. This finding was in line with studies in non-pregnant populations, where consumption of dairy and meats seemed to be less affected by the pandemic (15, 45). This might be due to the tight regulation of protein intake by biological control mechanisms known as “protein leverage theory” (46). Among those with changed intake, people tended to eat less meat and more dairy (15, 45). These patterns of change have been observed in pregnant individuals in pre-pandemic studies as well (3, 47). The main reason for intake changes of these food categories was altered craving, which is presumed to be more pregnancy-related than pandemic-related in our study. Craving dairy and aversion to meats has been described by previous research in pregnant individuals (3, 48–50). It is worth mentioning that the majority of our participants were at their second trimester at enrolment, and both frequency and intensity of pregnancy food cravings show a peak during the second trimester (51).

The greatest proportion of our participants ate about the same amount of canned foods and dried goods as pre-pandemic, which was in agreement with previous studies (45). But among those who changed their intake, more people increased their intake primarily because they could no longer go grocery shopping frequently. Spending more time on cooking was the top reason for decreased intake of this category but change in craving was also a main reason reported by the participants. Canned foods have been identified as one of the main aversion-prone categories during pregnancy (52).

Pre-pandemic studies of pregnant individuals have generally found increased intake of fruits and vegetables, mainly due to elevated craving and strive for a healthier diet (3, 47, 50). Change in craving was the top reason for increased intake of fruits and vegetables in our study as well.

Decreased consumption of fast-food and take-out/home delivery has been described by several previous studies (15, 45). Having more time to cook and prepare foods was the top reason for decreased intake of these two food categories in our study. Interestingly, as the stay-home restrictions were lifted in November in the USA, people reported eating more fast-food and take-out compared to the early phases of the pandemic (40). Craving was the main reason for increased intake; increased craving for ready-made meals during pregnancy has been describes by previous research (53, 54).

Sweets/snacks intake increased for 54.5% of our participants. This finding is in agreement with many studies during the pandemic with non-pregnant populations (11, 15, 45, 55). Increased snacking might be an attempt to soothe negative feelings like stress and boredom (56, 57). Pre-pandemic studies of pregnant individuals have also shown an increase in sweet foods intake mainly due to high craving (3, 50). Our findings were similar to these studies as we also observed that most participants increased their intake and that the main drive for this dietary change was increased craving. However, the percentage of people who increased their intake in our study was higher than the studies in non-pregnant populations during the pandemic and pregnant individuals in pre-pandemic times. This might suggest a cumulative effect of pregnancy-related craving and stress-related craving during the pandemic on increasing sweets intake.

### Changes in food consumption and mental health symptoms/resilience

The curvilinear pattern of association between food consumption and mental health indicators suggests that it was more the amount than the direction of change that was driving this association. This was especially true for fear of COVID-19, which was highest amongst those with either increased or decreased consumption of most food categories (except dairy). But this was less so the case for depressive and anxiety symptoms where the direction of change seemed to matter. Specifically, higher depressive and anxiety symptoms were associated with decreased intake of healthy food categories, such as vegetables/fruits and dairy, and also with higher intake of canned foods/dried goods, and either increased or decreased intake of the other relatively unhealthy food categories. This curvilinear pattern for fear has been reported by previous research too, with high fear of COVID-19 being associated with both increased and decreased frequency of eating meals and consumption of most food products (58). In a study among pregnant individuals, emotional eating was associated with increased cereals but decreased fish intake (26). Results from the previous studies regarding anxiety and depression are not consistent. Higher anxiety and depression scores were associated with increase in unhealthy foods intake in some studies (27, 45), increased intake of both healthy and unhealthy foods in one study (59), and restrained eating in another study (60). Stress-related eating can result in a paradox, in which there is an inconsistent direction of association between stress and eating. Stress severity, stress duration and individual differences might affect how people respond to fear or stress, anxiety, or depression (61).

### Strengths and limitations

This study had much strength including our sample size, participants from across Canada, data collection early in the pandemic and throughout the first year, and data available on various demographic, pandemic-related, and mental health factors that could contribute to dietary change.

Our study has some limitations. First, most participants self-identified as white, had a bachelor’s degree or above, and earned an annual household income over $70,000, which differs slightly from the Canadian female population of child-bearing age (62). Therefore, the results of this study might not generalize to populations with high socioeconomic risk. Second, our dietary questionnaire is not quantitative (i.e., we did not estimate actual consumption, just changes in consumption), which limits the conclusions that can be drawn. Third, neither dietary intake nor mental health symptoms are static throughout pregnancy. Collecting data on dietary changes only at the baseline survey precluded examination of how these dietary changes might have further changed within individuals over the course of their pregnancies. This might be especially relevant to factors such as changes in cravings, which were a main self-reported reason for changes in consumption of some food categories, because food cravings can come and go throughout pregnancy (51). Finally, there might be other factors that could play a role in how pregnant people change their diet for which we did not have data on (e.g., food and diet literacy, pre-existing eating disorders, and pre-existing mental health problems).

## Conclusion

Some pregnant individuals are more likely to change their diet during the pandemic due to socioeconomic risk factors, pandemic-related hardships, and health-related parameters. Particularly, mental health may be involved in how pregnant individuals change their intake of healthy and unhealthy food categories. Identifying these factors can prepare the healthcare system for future pandemic-like situations by informing what measures could be taken to protect this vulnerable population against dietary changes detrimental to their own and offspring’s health. The pandemic has also provided the opportunity for some healthy dietary practices like more cooking at home. This learning could be used to adjust working models even during non-pandemic times, in a way that allows people enough time to prepare healthy foods. Also, government financial support might have been effective in ensuring people have adequate access to their primary needs during hard times, as decreased affordability was not a main reason for dietary changes in our study. Taken together, the findings of our study might help with prevention of adverse effects of the pandemic-like situations on pregnant individuals’ dietary changes.

## Data availability statement

The raw data supporting the conclusions of this article will be made available by the authors, without undue reservation.

## Ethics statement

The current study was reviewed and approved by University of Calgary Conjoint Health Research Ethics Board (REB20-0500), Calgary, AB, Canada. The participants provided their written informed consent to participate in this study.

## Author contributions

EV-M, GG, and YW conceived the study. EV-M, JZ, and GB drafted the manuscript. EV-M, GG, YW, and MW contributed to the figures design, preparation, and edition. All authors contributed to the acquisition and analysis of data, critically reviewed the manuscript, gave final approval for all aspects of the work, agreed to be fully accountable for ensuring the integrity and accuracy of the work, and read and approved the final manuscript.

## Abbreviations

ANCOVAs: Analysis of covariance
ANOVA: Analysis of variance
BMI: Body mass index
CD-RISC 2: Connor-Davidson Resilience Scale
EPDS: Edinburgh Postpartum Depression Scale
FDR: False discovery rate
PdP: Pregnancy during the COVID-19 Pandemic
PROMIS: Patient-Reported Outcomes Measurement Information System
REDCap: Research Electronic Data Capture.
