# Supplementary material for: Dietary changes among pregnant individuals compared to pre-pandemic: A cross-sectional analysis of the Pregnancy during the COVID-19 Pandemic (PdP) study
Source: Front Nutr. 2022 Dec 1;9:997236. doi: 10.3389/fnut.2022.997236 (PMC9751429; doi:10.3389/fnut.2022.997236)
Supplement: Supplementary file 1 [file Table_1.DOCX]

| **Supplementary Table 1. Comparison of participants included in the analyses versus those who were not** | | | |
| --- | --- | --- | --- |
| **Variables** | **Participants included in the analyses (n=9870)** | **Participants not included in the analyses (n=980)** | ***P*** |
|  | ***Mean (SD)*** | |  |
| Age | 31.86 (4.39) | 31.24 (4.97) | <0.001^*^ |
| Gestational age at survey (week) | 20.53 (8.70) | 20.36 (8.56) | 0.550^*^ |
| Pre-BMI (kg/m2) | 26.24 (5.85) | 26.29 (5.88) | 0.793^*^ |
|  | ***N (%)*** | |  |
| Ethnicity |  |  |  |
| White (Caucasian) | 8239 (84.7) | 445 (82.7) | 0.764^†^ |
| Other minorities | 1494 (15.3) | 93 (17.3) |  |
| Education |  |  |  |
| Professional (MD, JD, DDS, ETC) | 453 (4.6) | 21 (3.0) | <0.001^†^ |
| Doctoral Degree (PhD) | 298 (3.0) | 12 (1.7) |  |
| Masters’ degree | 1800 (18.3) | 79 (11.2) |  |
| Bachelor’s degree | 3843 (39.1) | 239 (33.9) |  |
| Completed Trade/ Technical Degree | 2503 (25.5) | 242 (34.3) |  |
| Completed high school diploma | 808 (8.2) | 90 (12.8) |  |
| Less Than High School Diploma | 119 (1.2) | 22 (3.1) |  |
| Household income |  |  |  |
| ≥$200,000 | 952 (9.7) | 43 (7.9) | <0.001^†^ |
| $175,000-$199,999 | 624 (6.4) | 23 (4.2) |  |
| $150,000-$174,999 | 1086 (11.1) | 39 (7.1) |  |
| $125,000-$149,999 | 1286 (13.1) | 61 (11.2) |  |
| $100,000-$124,999 | 1844 (18.8) | 88 (16.1) |  |
| $70,000 – $99,9999 | 1949 (19.9) | 110 (20.1) |  |
| $40,000 – $69,9999 | 1296 (13.2) | 97 (17.7) |  |
| $20, 000 – $39,999 | 545 (5.6) | 52 (9.5) |  |
| <$20,000 | 218 (2.2) | 34 (6.2) |  |
| Job status |  |  |  |
| Woking full-time | 7515 (76.7) | 403 (72.5) | 0.006^†^ |
| Working part-time | 1116 (11.4) | 63 (11.3) |  |
| Unemployed /laid off | 206 (2.1) | 19 (3.4) |  |
| Looking for work | 111 (1.1) | 7 (1.3) |  |
| Keeping house/raising children | 848 (8.7) | 64 (11.5) |  |
| Retired | 2 (0.0) | 0 (0.0) |  |
| Marital status |  |  |  |
| Married/cohabiting | 9397 (95.7) | 652 (92.7) | 0.448^†^ |
| Other | 428 (4.3) | 51 (7.3) |  |
| ^*^ ANOVA  ^†^ Pearson Chi-square |  |  |  |
